# Risk factors for substantial weight retention at 1 year postpartum: evidence from a German birth cohort study (KUNO-Kids)

**DOI:** 10.1007/s00404-024-07795-6

**Published:** 2024-11-27

**Authors:** S. Quaderer, S. Brandstetter, A. Köninger, M. Melter, M. Kabesch, C. Apfelbacher, S. Fill Malfertheiner, KUNO-Kids Study Group, Andreas Ambrosch, Petra A Arndt, Andrea Baessler, Mark Berneburg, Stephan Böse-O’Reilly, Romuald Brunner, Sara Fill Malfertheiner, André Franke, Sebastian Häusler, Iris Heid, Stefanie Heinze, Wolfgang Högler, Sebastian Kerzel, Michael Koller, Michael Leitzmann, Áine Lennon, David Rothfuß, Wolfgang Rösch, Bianca Schaub, Stephan Weidinger, Sven Wellmann

**Affiliations:** 1 Department of Gynecology and Obstetrics, Hospital St. Hedwig of the Order of St. John, University Medical Center Regensburg, Steinmetzstrasse 1‑3, 93049 Regensburg, Germany; 2 University Children’s Hospital Regensburg (KUNO), Hospital St. Hedwig of the Order of St. John, Steinmetzstrasse 1‑3, 93049 Regensburg, Germany; 3 The Research and Development Campus Regensburg (WECARE), Hospital St. Hedwig of the Order of St. John, University Medical Center Regensburg, Steinmetzstrasse 1‑3, 93049 Regensburg, Germany; 4 Institute of Social Medicine and Health Systems Research, Otto Von Guericke University Magdeburg, Leipziger Strasse 44, 39120 Magdeburg, Germany

**Keywords:** Postpartum weigh retention, Substantial weight retention, Postpartum, Overweight, Obesity

## Abstract

**Purpose:**

Postpartum weight retention (PPWR) increases the risk of overweight and obesity. This study aims to identify risk factors for substantial weight retention (≥ 5 kg) at 1 year postpartum.

**Methods:**

Data were obtained from *N* = 747 mothers participating in the KUNO-Kids birth cohort study. The following variables were analyzed: sociodemographic variables, pre-pregnancy body mass index, postpartum weight retention at 6 months, gestational weight gain, parity, breastfeeding, mode of delivery, gestational diabetes mellitus, physical activity, diet, alcohol consumption, smoking, sleep, and depression. Variables that showed an association of *p* < 0.2 with substantial postpartum weight retention (SPPWR) in univariable logistic regression analyses were included in the multivariable logistic regression analysis. Statistical analyses were performed using IBM SPSS.28.

**Results:**

One year after delivery, mean PPWR was 1.5 kg (SD 5.2 kg), and 21.6% of the women had SPPWR. The multivariable logistic regression model showed a significant negative association of SPPWR with an intermediate educational status compared to a low educational status (OR = 0.27 [95% CI 0.11–0.69]). In addition, PPWR at 6 months was positively associated with SPPWR (OR = 1.55 [95% CI 1.43–1.69]) at 1 year. None of the other associations reached statistical significance.

**Conclusion:**

Postpartum weight retention may lead to weight gain. Losing weight in the first few months after delivery may prevent substantial postpartum weight retention. Women of low education may particularly benefit from weight loss support.

## What does this study add to the clinical work

**Table:** 

Postpartum weight retention may lead to weight gain. Losing weight in the first few months after delivery may prevent substantial postpartum weight retention. Women of low education may particularly benefit from weight loss support.	

## Introduction

Obesity is associated with an increased risk of insulin resistance, dyslipidemia, hypertension, and cardiovascular disease, which may lead to life-threatening events such as stroke, myocardial infarction, and heart failure [1–4]. For women of reproductive age, obesity is associated with reduced fertility and higher rates of complications during pregnancy including gestational diabetes mellitus, preeclampsia, gestational hypertension, depression, cesarean section and surgical site infection [5, 6]. In addition, children of obese mothers are more likely to be born prematurely, suffer from fetal defects or specific congenital anomalies, and even die perinatally [7]. Obesity rates have increased worldwide in the past decades [8, 9].

One major factor provoking weight gain in women is pregnancy [10]. Several studies have not only shown temporary gestational weight gain during pregnancy but also long-term weight gain with postpartum weight retention (PPWR) at one year after delivery ranging from 0.5 kg to 2.6 kg [11–14]. While the findings of mean PPWR suggest that the effect on maternal weight is rather small, the percentage of women with substantial postpartum weight retention (SPPWR), defined as PPWR of at least 5 kg, is clearly not negligible. According to several previous studies, up to 25.6% of mothers have retained 5 kg or more at one year postpartum [11–14]. Therefore, SPPWR can easily induce a relevant change in weight and body mass index (BMI).

Numerous studies have examined maternal weight changes in the postpartum period and the factors that may influence such changes. According to the literature, one important factor associated with PPWR is gestational weight gain (GWG). Several studies have described an association between PPWR and GWG that exceeds the recommendations established by the Institute of Medicine (IOM) [15–18]. In contrast, numerous studies suggest that PPWR is inversely associated with physical activity [12, 19–21], breastfeeding [22, 23] and pre-pregnancy BMI [16, 24, 25]. However, the association with other factors remains unclear, including mode of delivery [26, 27], parity [28], and depression [24, 29, 30]. Therefore, the aim of this study was to analyze a wide range of variables for their association with SPPWR at 1 year postpartum*,* using data from a large German birth cohort study (KUNO-Kids).

## Methods

### Study design and setting

Data were obtained from the KUNO-Kids study, a prospective multi-purpose cohort study of children born at the Hospital St. Hedwig, Regensburg, Germany, and their families [31]. The Hospital St. Hedwig is a university obstetrics and children’s hospital in southern Germany with approximately 3600 births per year. Data were collected within 3 days after delivery when mothers were interviewed by study personnel in the hospital and filled in questionnaires. Information on the pre-pregnancy and peripartum period was collected retrospectively.

Follow-up questionnaires are sent to the family at 4 weeks, at 6 months, and at 1 year postpartum.

### Participants and data collection

Criteria for participation in the KUNO-Kids study are written informed consent from the mothers, who need to be at least 18 years of age and fluent in German. For this study, we collected data from 3100 mothers and their children born between June 2015 and March 2020. The inclusion criteria for this analysis were available data on weight before pregnancy and at the 1-year follow-up, gestational age at birth within gestational weeks 37 + 0 and 41 + 6, singleton pregnancy, and no additional pregnancy at 1 year postpartum. Women were asked whether their weight was measured or estimated. Only women who reported that their weight was measured were included.

### Variables and measurement

#### Outcome and predictor variables

The primary outcome of this study is SPPWR at 1 year postpartum. SPPWR was defined as PPWR of at least 5 kg and evaluated as a dichotomous variable. PPWR was calculated by subtracting the weight at 12 months postpartum from the pre-pregnancy weight.

#### Sociodemographic variables

Sociodemographic data on education, migration background, and marital status were recorded in the baseline interview. Information on maternal age was taken from the baseline information of the KUNO-Kids study. Educational status was categorized as “low” (CASMIN 1), “intermediate” (CASMIN 2), and “high” (CASMIN 3) according to the “Comparative Analysis of Social Mobility in Industrial Nations” (CASMIN) classification [32]. Migration background was assumed if the mother or one of the mother’s parents was born in a country other than Germany.

#### Weight-related variables

Information on the mother’s height, pre-pregnancy weight, and gestational weight gain was obtained at the baseline interview. Data on the postpartum weight were collected in the 6 month and 1 year follow-up questionnaires. The BMI was calculated by weight in kilograms divided by the square of height in meters and categorized into four groups: underweight (< 18.5 kg/m^2^), normal weight (18.5–24.9 kg/m^2^), overweight (25.0–29.9 kg/m^2^), and obese (≥ 30.0 kg/m^2^) [33]. Gestational weight gain (GWG) was defined as the maximum weight gained during pregnancy compared with pre-pregnancy weight. GWG was categorized as within, below, or above the 2009 IOM recommendations for each BMI category [34]. These recommendations are as followed: 12.5–18 kg for underweight, 11.5–16 kg for normal, 7–11,5 kg for overweight and 5–9 kg for obese women.

#### Obstetric variables

Obstetric factors such as the mode of delivery, parity, gestational diabetes mellitus and postpartum midwife consultation were assessed as dichotomous variables. Breastfeeding was defined as exclusive breastfeeding for at least four months, meaning that the child received only breast milk [35].

#### Lifestyle-related variables

Physical activity 1 year after delivery was assessed as a dichotomous variable. In the category “as recommended by the WHO or more”, we included the answer “2–4 h per week or more” in the past 6 months. This time period refers to the WHO recommendation of 150 min of moderate-intensity physical activity per week [36].

Diet was considered healthy if the mother reported eating fruits or vegetables almost every day or several times per week. In addition, smoking and alcohol consumption at 1 year postpartum were assessed as dichotomous variables. For alcohol, we evaluated whether there was risky alcohol consumption from 6 months to 1 year postpartum. This refers to the recommendation that women should not consume more than 12 g of alcohol per day [37].

#### Psychosocial variables

Maternal sleep quality was assessed using the Pittsburgh Sleep Quality Index with questions from the 1-year questionnaire. This questionnaire includes information on the subjective sleep quality, sleep latency, sleep duration, habitual sleep efficiency, sleep disturbances, use of sleep medications, and daytime dysfunction. A global score of > 5 indicates poor sleep quality [38]. Mental health was evaluated using the PHQ-D questionnaire which is used as a screening tool for depressive disorders [39]. Major depression can be assumed at a score of at least 10 points out of 27. In this study, depression was assumed if the score for major depression was positive at either 6 months or 1 year postpartum, or both.

### Statistical analysis

We checked for plausibility and excluded any implausible data. To describe the socio-demographic data of the sample, means with standard deviations were calculated for continuous data and frequencies with percentages for categorical data. The outcome variable SPPWR is presented as frequencies with percentages. Univariable logistic regression analysis was used to evaluate the association between the selected variables and SPPWR. Predictor variables that showed an association with *p* < 0.2 in univariable logistic regression analysis were included in multivariable logistic regression analysis. We checked the multivariable model for collinearity of the predictor variables.

Statistical analyses were performed with IBM SPSS.28.

## Results

### Sample description

**Fig. 1:**
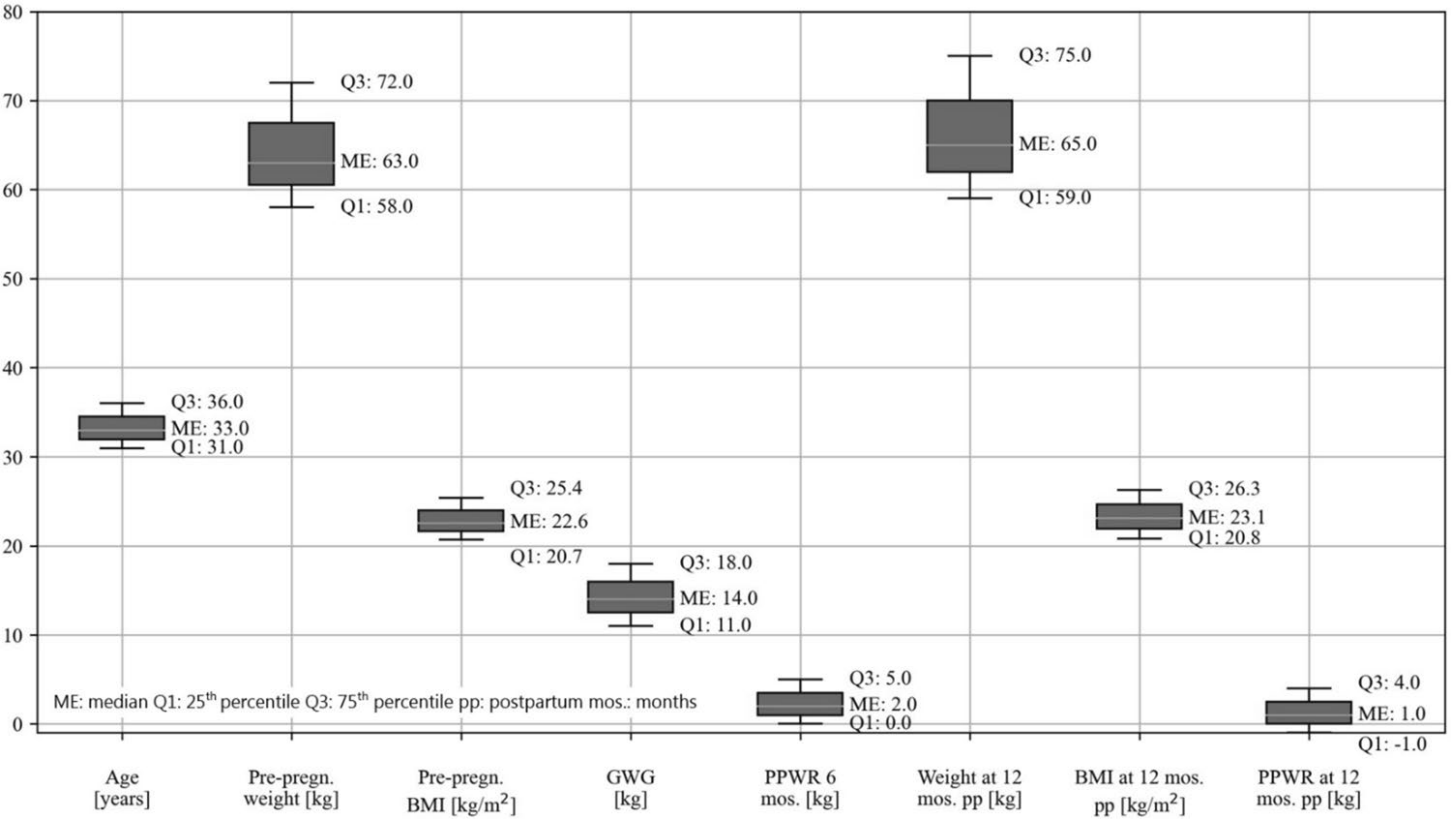
Characteristics of the sample—median and interquartile range

The characteristics of the study sample are listed in Table 1. 3100 women gave their written informed consent and were included in this study. In a second step, we excluded women who did not meet the following criteria for this analysis: available data on weight before pregnancy and at the 1-year follow-up, gestational age at birth within gestational weeks 37 + 0 and 41 + 6, singleton pregnancy, and no additional pregnancy at 1 year postpartum. After comparing weight data that women had reported as “estimated” and “measured,” we excluded all data from participants with “estimated” weight. In the end, 747 women met the inclusion criteria for this study (Fig.1).

**Table 1:** Characteristics of the sample Mothers (total *N* = 747)

Variable	*N*	Value
Sociodemographic factors		
Age (years) *M (SD)*	740	33.2 (4.1)
Educational status	731	
CASMIN 1 [low education] *n (%)*		52 (7.1)
CASMIN 2 [intermediate education] *n (%)*		319 (43.6)
CASMIN 3 [high education] *n (%)*		360 (49.2)
Marital status	737	
Married or living with partner *n (%)*		727 (98.6)
Married, not living with partner; single; divorced or widowed *n (%)*		10 (1.4)
Migration background *n (%)*	747	101 (14.2)
Weight-related factors		
Pre-pregnancy weight (kg) *M (SD)*	747	66.6 (13.6)
Pre-pregnancy BMI (kg/m^2^) *M (SD)*	747	23.7 (4.6)
Underweight *n (%)*		33 (4.4)
Normal weight *n (%)*		509 (68.1)
Overweight *n (%)*		136 (18.2)
Obese *n (%)*		69 (9.2)
Gestational weight gain (GWG) (kg) *M (SD)*	742	14.6 (5.3)
< IOM recommended *n (%)*		154 (20.8)
= IOM recommended *n (%)*		286 (38.5)
> IOM recommended *n (%)*		302 (40.7)
PPWR 6 months (kg) *M (SD)*	729	2.8 (5.1)
Weight at 12 months pp (kg) *M (SD)*	747	68.1 (14.2)
BMI at 12 months pp (kg/m^2^) *M (SD)*	747	24.2 (4.9)
Underweight *n (%)*		37 (5.0)
Normal weight *n (%)*		452 (60.5)
Overweight *n (%)*		169 (22.6)
Obese *n (%)*		89 (11.9)
PPWR at 12 months pp (kg) *M (SD)*	747	1.5 (5.2)
SPPWR at 12 months pp *n (%)*	747	161 (21.6)
Obstetric factors		
Parity (primiparous) *n (%)*	747	436 (58.4)
Full breastfeeding for ≥ 4 months *n (%)*	709	484 (68.3)
Mode of delivery (Cesarean section) *n (%)*	730	218 (29.9)
Gestational diabetes mellitus *n (%)*	739	118 (16.0)
Support by midwife in the pp period *n (%)*	746	722 (96.8)
Lifestyle-related factors		
Physical activity (≥ 2–4 h/week) from 6–12 months pp *n (%)*	746	172 (23.1)
Healthy diet at 12 months pp *n (%)*	731	386 (52.8)
Probiotics within 12 months pp *n (%)*	746	63 (8.4)
Alcohol consumption at risk from 6–12 months pp *n (%)*	724	40 (5.5)
Smoking at 12 months pp *n (%)*	740	63 (8.5)
Psychosocial factors		
Poor sleep (Pittsburgh Sleep Quality Index) *n (%)*	644	277 (43.0)
Depression (PHQ-9 Depression Score) *n (%)*	746	100 (13.4)

*N* Number of women included n: number of women for this specific item, *M* Mean value, *SD* Standard deviation, *pp* Postpartum

Mean age was 33.2 years (standard deviation [SD], 4.1). 98.6% of the women were married or living with their partner; 14.2% had a migration background. According to the CASMIN classification, 49.2% of the participants had a high, 43.6% an intermediate, and 7.1% a low educational status.

Mean pre-pregnancy weight was 66.6 kg (SD 13.6) compared to 68.1 kg (SD 14.2) at one year postpartum. Mean pre-pregnancy BMI was 23.7 kg/m^2^ (SD 4.6); 18.2% of the women were classified as overweight and 9.2% as obese. In comparison, mean BMI at one year postpartum was 24.2 kg/m^2^ (SD 4.92); 22.6% of the women were classified as overweight and 11.9% as obese. Mean PPWR at 1 year postpartum was 1.5 kg (SD 5.2 kg), and 21.6% of the women had SPPWR.

### Univariable regression analyses

Univariable regression analyses showed a positive association of SPPWR with PPWR at 6 months (odds ratio (OR) = 1.53 [95% confidence interval (CI) 1.43–1.64]), a pre-pregnancy BMI (kg/m^2^) (OR = 1.07 [95% CI 1.03–1.11]) that exceeded the IOM guidelines for gestational weight gain (OR = 4.49 [95% CI 2.93—6.89]), and primiparity (OR = 1.55 [95% CI 1.10–2.23]). In contrast, SPPWR was inversely associated with high (OR = 0.37 [95% CI 0.20–0.67]) and intermediate educational status (OR = 0.32 [95% CI 0.17–0.59]) compared to low educational status, physical activity as recommended by the WHO or more (OR = 0.52 [95% CI 0.32–0.83]), and a healthy diet (OR = 0.52 [95% CI 0.36–0.75].

We found no significant association between SPPWR and exclusive breastfeeding for at least 4 months, postpartum midwife consultation, maternal age, migration background, marital status, mode of delivery, gestational diabetes mellitus, alcohol consumption, smoking, poor sleep, and depression. The results are shown in Table 2.

**Table 2:** Associations between SPPWR and potential risk factors:results of univariable logistic regression analysis Mothers (total *N* = 747), SPPWR (yes/no)

Variable	Reference category	Odds ratio (95% Confidence interval)	*P*-value
***Sociodemographic factors***			
Age (years)		1.01(0.97; 1.05)	0.713
Educational status			
CASMIN 1 [low education]	Reference		
**CASMIN 2 [intermediate education]**	**Compared to CASMIN 1**	**0.32(0.17; 0.59)**	**0.000**
**CASMIN 3 [high education]**	**Compared to CASMIN 1**	**0.37(0.20; 0.67)**	**0.001**
Married or living with partner		0.41(0.12; 1.48)	0.175
Migration background		1.18(0.72; 1.94)	0.518
Weight-related factors			
**Pre-pregnancy BMI (kg/m**^**2**^**)**		**1.07(1.03; 1.11)**	** < 0.001**
**PPWR 6 months (kg)**		**1.53(1.43; 1.64)**	** < 0.001**
Gestational weight gain (kg)			
< IOM recommended	Compared to IOM recommendations	0.68(0.35; 1.34)	0.266
= IOM recommended	Reference		
> IOM recommended	Compared to IOM recommendations	**4.49(2.93; 6.89)**	** < 0.001**
Obstetric factors			
**Primiparity**		**1.55(1.07; 2.23)**	**0.019**
Full breastfeeding for ≥ 4 months		0.72(0.50; 1.05)	0.086
Mode of delivery: Cesarean section		1.16(0.79; 1.69)	0.454
Gestational diabetes mellitus		0.85(0.52; 1.39)	0.510
Support by midwife in the pp period		0.53(0.22; 1.27)	0.155
Lifestyle-related factors			
**Physical activity (≥ 2–4 h/week) from 6–12 months pp**		**0.52(0.32; 0.83)**	**0.006**
**Healthy diet at 12 months pp**		**0.52(0.36; 0.75)**	** < 0.001**
Probiotics within 12 months pp		0.58(0.28; 1.21)	0.146
Alcohol consumption at risk from 6–12 months pp		0.76(0.33; 1.74)	0.510
Smoking at 12 months pp		1.51(0.85; 2.68)	0.164
Psychosocial factors			
Poor sleep (Pittsburgh Sleep Quality Index)		1.15(0.79; 1.68)	0.466
Depression (PHQ-9 Depression Score)		1.11(0.67; 1.83)	0.685

*N* Number of women included, *pp* Postpartum

### Multivariable regression analysis

The multivariable logistic regression model showed a significant negative association for SPPWR and intermediate educational status compared to low educational status (OR = 0.27 [95%-CI 0.11–0.69]). Furthermore, PPWR at 6 months was positively associated with SPPWR (OR = 1.55 [95%-CI 1.43–1.69]). The data did not show a significant association for any other variable. The results are shown in Table 3.

**Table 3:** Associations between SPPWR and potential risk factors: results of multivariable logistic regression analysis Mothers (total *N* = 651), SPPWR (yes/no)

Variable	Reference category	Odds Ratio 95% Confidence interval	*P*-value
Sociodemographic factors			
Educational status			
CASMIN 1 [low education]	Reference		
**CASMIN 2 [moderate education]**	**Compared to CASMIN 1**	**0.27(0.11; 0.69)**	**0.006**
CASMIN 3 [high education]	Compared to CASMIN 1	0.55(0.22; 1.41)	0.214
Married or living with partner		1.02(0.13; 8.08)	0.984
***Weight-related factors***			
Prepregnancy BMI (kg/m^2^)		1.03(0.97; 1.10)	0.359
**PPWR 6 months (kg)**		**1.55(1.43; 1.69)**	** < 0.001**
Gestational weight gain (kg)			
< IOM-recommended	Compared to IOM recommendations	1.16(0.48; 2.83)	0.743
= IOM-recommended	Reference		
> IOM-recommended	Compared to IOM recommendations	1.31(0.70; 2.46)	0.396
Obstetric factors			
Primiparity		1.69(0.98; 2.93)	0.060
Full Breastfeeding for ≥ 4 months		1.12(0.62; 2.02)	0.710
Support by Midwife in the pp period		0.58(0.13; 2.51)	0.467
Lifestyle-related factors			
Physical activity (≥ 2–4 h/week) from 6 to 12 months pp		0.70(0.37; 1.33)	0.28
Healthy nutrition at 12 months pp		0.63(0.37; 1.08)	0.09
Probiotics within 12 months pp		0.51(0.18; 1.43)	0.20
Smoking at 12 months pp		0.76(0.30; 1.97)	0.58

*N* Number of women included, *pp* Postpartum

## Discussion

This study examined potential risk factors for SPPWR at 1 year postpartum. 21.6% of the participating women had SPPWR at 1 year postpartum, and mean PPWR was 1.5 kg (SD 5.2). In the multivariable regression analysis, only education and postpartum weight retention were significantly associated with SPPWR.

Our results for SPPWR and PPWR are consistent with the findings of previous research. In the literature, SPPWR ranges from 12.0% to 25.6% and PPWR from 0.5 kg to 2.6 kg [11–14]. Our findings suggest that a higher PPWR at 6 months increases the risk of retaining 5 kg or more at 1 year postpartum. This shows that women who do not lose pregnancy weight in the first 6 months after delivery have a higher risk of not regaining their pre-pregnancy weight. In addition, we found a lower risk of SPPWR for intermediate education compared with low education. Consistent with our findings, previous research reported a higher risk of SPPWR for mothers with low education [11, 17, 24]. This association was not observed for high education compared to low education which appears to be implausible. For the education groups, the sample size may have been too small to detect an association, or the effect size may not have been large enough.

There was no association between age and SPPWR, but this association is controversially discussed in the literature: Pedersen and Siega-Riz reported a lower PPWR in women with increasing age, whereas the findings of Levine were consistent with our results [24, 40, 41].

Furthermore, we found no significant associations between SPPWR and migration background. To our knowledge, this factor has rarely been examined, although several studies have reported a positive association for certain ethnicities [17, 40].

Literature reports that being married seems to reduce the risk of PPWR [29, 42]. We could not find an association. This might be biased by the small number of women being single or not living with their partner in our study group.

GWG was not significantly associated with SPPWR. This finding contradicts previous research. Several studies have found GWG to be one of the strongest predictors of PPWR [15, 16, 18, 23–25, 30, 43, 44]. In our research, exceeding the IOM-guideline for GWG has been positively associated with SPPWR in the univariable analysis. This association lost significance in multivariable analysis.

Several studies have shown that as BMI increases, PPWR seems to decrease [14, 16, 24, 25, 40]. We could not detect a significant association in multivariable analysis.

Another frequently examined factor is breastfeeding. Several authors have reported that exclusive breastfeeding or a long period of breastfeeding are inversely associated with PPWR [16, 20, 22–24, 43, 45]. In our study, breastfeeding was inversely associated with SPPWR in univariable analysis, but lost significance in multivariable analysis.

Previous studies have shown inconsistent results for the mode of delivery. Consistent with our results, Kapinos found no association between cesarean section or vaginal delivery and weight at the beginning of the second pregnancy [26]. In contrast, Legro reported a higher possibility of PPWR of 10 pounds or more at 1 year postpartum for delivery by cesarean section [27].

Hollis found that primiparous women seem to be more likely to retain weight at six months postpartum [16]. We did not find any association.

To our knowledge, no study has analyzed the association between gestational diabetes mellitus and PPWR. We found no significant association in our study.

Another important factor related to weight is nutrition. Ng et al. showed that women were more likely to retain weight if they ate three or fewer servings of fruit or vegetables per day [20]. Boghossian found that the total energy intake was more important than a specific diet [42]. In our multivariable model, we found no significant association between daily fruit and vegetable intake and SPPWR. It seems obvious that diet is associated with weight maintenance and retention. Nevertheless, we found no significant association. One reason may be that our parameter “nutrition” does not reflect actual dietary intake. We analyzed regular vegetable and fruit intake as an approximation of a healthy diet. Due to the limitations of our study, food composition or total calorie intake were not considered in this paper.

Several studies reported that physical activity was inversely associated with PPWR or SPPWR [11, 12, 19–21, 46]. In our study, physical activity was associated with a decreased risk of SPPWR in univariable logistic regression analysis but lost significance in the multivariable model.

Smoking was not associated with SPPWR. In a study by Levine, smoking was associated with decreased PPWR when started after pregnancy, whereas Herring found no association [29, 41]. We also did not find any significant association with alcohol. Actual alcohol consumption may differ from self-reported answers. Because alcohol consumption and smoking are perceived negatively by society, women may deny smoking or report a lower amount of alcohol than they actually consume. Moreover, the sample size may be too small to detect an association between SPPWR and smoking and alcohol.

Depression has also been a subject of research in recent years with inconsistent findings [24, 29, 30, 47]. Our data did not show any association.

There was no significant association with sleep in this study. Several studies suggest that PPWR might be inversely associated with sleep duration [40, 48, 49].

It might be possible that weight is influenced by the intake of drugs or contraceptives. Due to study limitations, we did not consider the association with SPPWR.

### Strengths and limitations

We observed an overrepresentation of well-educated and socioeconomically advantaged families, which can be partly explained by the settings of this study: the exclusion of mothers under the age of 18 years or with inadequate language skills resulted in the exclusion of a specific part of the population. In addition, well-educated adults are more likely to participate in a study because they may be more interested in scientific research and be more aware of its importance. This effect, which is difficult to avoid, has been seen in other related birth cohort studies [50, 51]. Another common problem in longitudinal birth cohort studies is loss to follow-up. The KUNO-Kids study shows that a significant percentage of families drop out between the birth of a child and the 1-year follow-up. This effect not only leads to the loss of participants but may also limit the validity of a study in case of differential loss to follow-up. In the KUNO-Kids study, this effect leads to an increasing overrepresentation of well-educated families [31].

According to the National Obstetrics Evaluation in Germany from 2017, 15.7% of women were considered obese at the beginning of the pregnancy [52]. In contrast to this, before pregnancy only 9.2% of women in our study population fall into this category. This discrepancy could introduce a bias and potentially alter the results. One possible explanation for the significantly lower participation of obese individuals in the study could be related to education level. Being less educated appears to be associated with a higher risk of obesity [53]. Thus, the previously mentioned effect of an overrepresentation of well-educated participants may account for this discrepancy.

Information on weight was self-reported by the mothers. Due to study limitations, it was not possible to have the mothers’ weight measured by the study personnel. Women were asked to indicate whether their self-reported weight was measured or estimated. We compared the two groups in a sensitivity analysis and found diverging results for some parameters. Therefore, we decided to include only women who reported that their weight had been measured. However, weight can be a sensitive issue for some people, so self-reported weight may differ from the actual weight. When analyzing 15 studies on the comparability of self-reported and reference standard measurements, Rubeis found that the difference was rather small. For this reason, self-reported weight is suggested to be a valid measure [54].

An obvious strength of the KUNO-Kids study is its large number of participants as 747 women were included in this research project. Another unique feature is the holistic approach of the study, which made it possible to analyze a wide range of variables and examine SPPWR in a broader context.

## Conclusion

Women with higher weight retention at 6 months postpartum are at risk for retaining 5 kg or more at 12 months after delivery. Losing weight within the first months postpartum may prevent substantial postpartum weight retention. Women of low education may benefit particularly from weight loss support.

## Data Availability

Data and materials and the datasets used and/or analyzed for this paper are available from the corresponding author on reasonable request.
